# Supplementary material for: Soyasapogenol C from Fermented Soybean (Glycine Max) Acting as a Novel AMPK/PPARα Dual Activator Ameliorates Hepatic Steatosis: A Novel SANDA Methodology
Source: Int J Mol Sci. 2022 May 13;23(10):5468. doi: 10.3390/ijms23105468 (PMC9141180; doi:10.3390/ijms23105468)
Supplement: Supplementary file 1 [file ijms-23-05468-s001.zip › ijms-1684780-supplementary.pdf]

Supplementary Materials for Article

Soyasapogenol C from Fermented Soybean (*Glycine Max*) Acting as a Novel AMPK/PPARα Dual Activator Ameliorates Hepatic Steatosis: A Novel SANDA Methodology

Radha Arulkumar [1], Hee Jin Jung [2], Sang Gyun Noh [1] and Hae Young Chung [1,2,*]

[1] Interdisciplinary Research Program of Bioinformatics and Longevity Science, Pusan National University, Busan 46241, Korea; radhuspn@gmail.com (R.A.); rskrsk92@naver.com (S.G.N.)
[2] Department of Pharmacy, College of Pharmacy, Pusan National University, Busan 46241, Korea; hjjung2046@pusan.ac.kr
* Correspondence: hyjung@pusan.ac.kr; Tel.: 82-51-510-2814

Supplementary Materials:

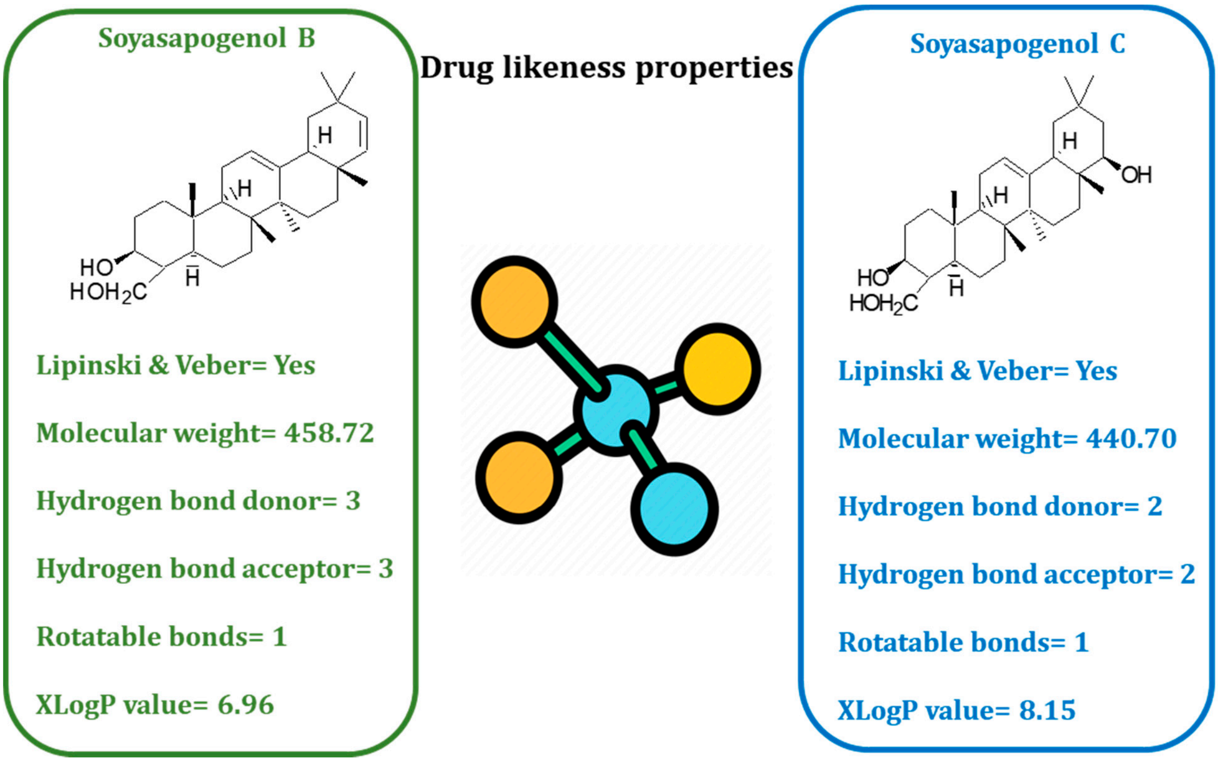

Figure S1. The structural similarity evaluation of soyasapogenol B and soyasapogenol C.

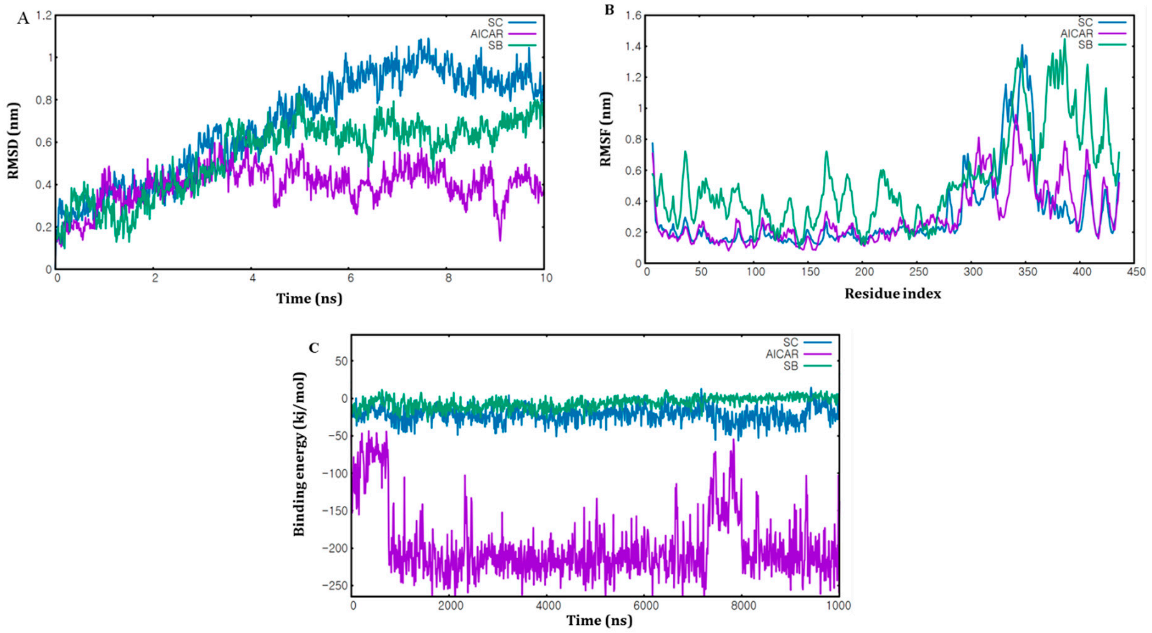

Figure S2. Molecular dynamic simulation: (A) RMSD plots between soyasapogenol C (blue), soyasapogenol B (green), and AICAR (purple) with AMPK; (B) RMSF plots between soyasapogenol C (blue), soyasapogenol B (green), and AICAR (purple) with AMPK; (C) Binding interaction plots between soyasapogenol C (blue), soyasapogenol B (green), and AICAR (purple) with AMPK at 10ns. *SC—soyasapogenol C; SB—soyasapogenol B; AICAR—5-Aminoimidazole-4-carboxamide ribonucleotide.

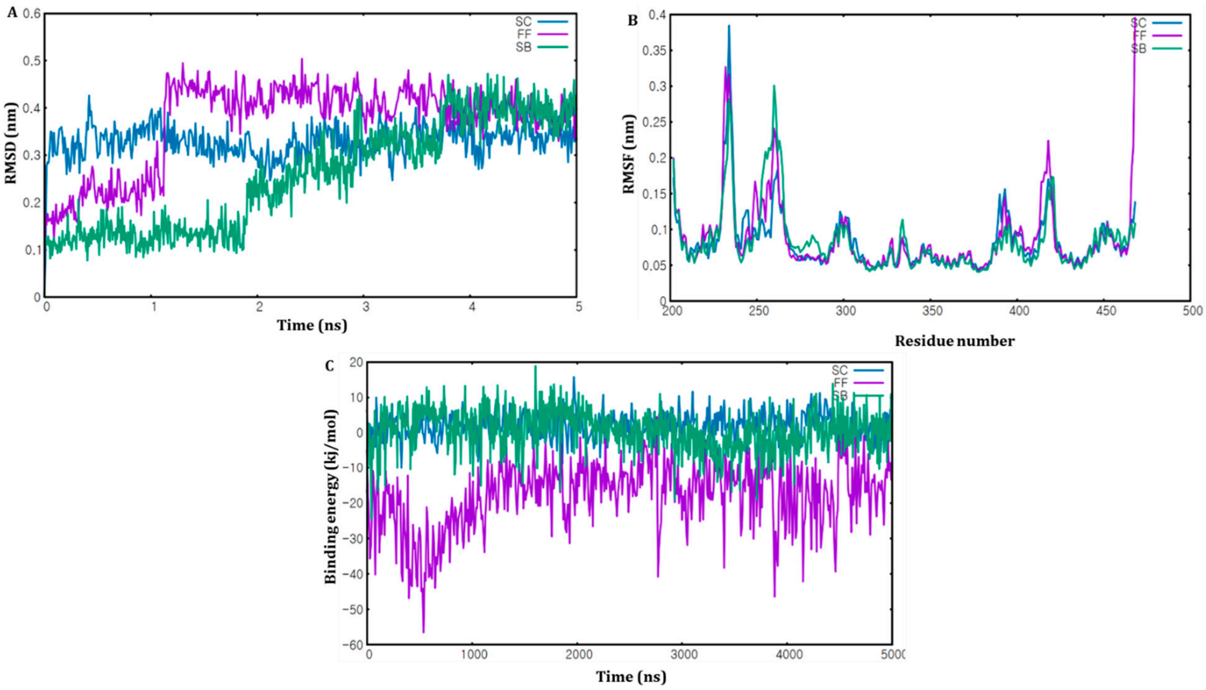

Figure S3. Molecular dynamic simulation: (A) RMSD plots between soyasapogenol C (blue), soyasapogenol B (green), and fenofibrate (purple) with PPARα. (B) RMSF plots between soyasapogenol C (blue), soyasapogenol B (green), and fenofibrate (purple) with PPARα at 5 ns. (C) Binding interaction plots between soyasapogenol C (blue), soyasapogenol B (green), and fenofibrate (purple) with PPARα at 5ns. * SC—soyasapogenol C; SB—soyasapogenol B; FF—fenofibrate.

Table S1. Group B soyasaponin, soyasaponin, DDMP saponin, and soyasaponin aglycones present in fermented soybean Cheonggukjang, and their activities.

Serial. No	Types	Compounds	Effects
1	Group B soyasaponin	Soyasaponin 1	Anti-inflammatory, anti-carcinogenic, hepatoprotective, cardiovascular protective, antimicrobial, anti-oxidative, adjuvant kidney protective
2		Soyasaponin II	Hepatoprotective, cardiovascular protective, anti-viral
3		Soyasaponin III	Anti-inflammatory, anti-carcinogenic, hepatoprotective
4		Soyasaponin IV	Anti-carcinogenic, cardiovascular protective
5		Soyasaponin V	
6		Soyasaponin VI	
7	Soyasaponin	Dehydrosoyasaponin I	Potent and reversible calcium-activated potassium (maxi-K) channels activator
8	Soyasaponin aglycone	Soyasapogenol A	Anti-carcinogenic, hepatoprotective, anti-viral
9		Soyasapogenol B	Anti-inflammatory, anti-mutagenic, anti-carcinogenic, hepatoprotective, anti-viral
10		Soyasapogenol C	Anti-Inflammatory, anti-viral
11		Soyasapogenol D	
12	DDMP group of soyasaponin	Soyasaponin αg	Antioxidant, anti-obesity, anti-tumorigenic, anti-hypertension, anti-arcopenia, skin whitening, immune modulation, sympathetic nerve activity, anti-diabetic activity
13		Soyasaponin βa	
14		Soyasaponin γa	
15		Soyasaponin yg	

Table S2. Docking score of Soyasaponin B group, soyasaponin, DDMP saponin, and soyasaponin aglycones present in fermented soybean Cheonggukjang using Auto Dock Vina and AutoDock4.

	AMPK		LKB1		PPARα	
Compound	AutoDock Vina	AutoDock4	AutoDock Vina	AutoDock4	AutoDock Vina	AutoDock4
Control	-5.7	-10.81	-7.4	-7.22	-6.7	-7.81
Soyasaponin I	2.6	-3.69	0.6	-10.24	12.8	52.74
Soyasaponin II	0.6	0.66	-3.4	-3.95	19.1	68.81
Soyasaponin III	19.2	416.51	35.6	376.24	122.8	2.37E+03
Soyasaponin IV	-3.3	-10.31	1	-10.85	6.8	19.82
Soyasaponin V	25.6	337.57	26.7	218.58	88.8	6.39E+03
Soyasaponin VI	6.4	86.93	1.2	31.8	25	236.48
Soyasapogenol A	-3.3	-8.22	-8.2	-10.35	3.5	-1.84
Soyasapogenol B	-3.7	-7.68	-8.8	-9.3	2	-5.82
Soyasapogenol C	-7.2	-10.24	-9.4	-8.97	4.2	-8.95
Soyasapogenol D	-6.4	-10.98	-8.2	-10.16	4.2	-2.12
Soyasaponin αg	17.6	362.34	8.4	216.29	26.2	271.11
Soyasaponin βa	9.3	102.16	0.2	138.88	21.5	385.91
Soyasaponin γa	-4.1	6.67	-6.2	-4.14	3.8	20.45
Soyasaponin γg	-0.9	1.62	-0.8	-7.13	-1	29.62
Dehydrosoyasaponin I	4.7	-4.62	2	-13.05	14	27.21

Table S3. In silico docking score of active compounds soyasapogenol C, soyasapogenol B, and control compounds AICAR (AMPK) and fenofibrate (PPARα) with targets AMPK and PPARα, using four different docking tools.

Compounds	AMPK (PDB ID: 2Y94)			
	Auto Dock Vina	AutoDock4	Swiss Dock	LeDock
AICAR*(Control)	-6	-7.54	-9.01	-6.01
Soyasapogenol B	-3.7	-7.68	-7.39	-4.67
Soyasapogenol C	-7.2	-10.24	-6.96	-4.64

*AICAR- 5-Aminoimidazole-4-carboxamide ribonucleotide

Compounds	PPARα (PDB ID: 1K7L)			
	Auto Dock Vina	AutoDock4	Swiss Dock	LeDock
Fenofibrate (Control)	-7.4	-9.67	-8.59	-3.67
Soyasapogenol B	2	-5.82	-5.47	-2.31
Soyasapogenol C	4.2	-8.95	-7.91	-2.17

Table S4. The binding energy and interacting residues obtained by calculating the molecular docking data of AMPK and PPARα with controls and soyasapogenol C, using Auto Dock 4.2.

Compounds	Binding energy (kcal/mol)	Intermolecular energy (kcal/mol)	Common residues
AMPK			
Soyasapogenol C	-10.24	-11.16	VAL24, GLY25, VAL30, LYS45, MET93, GLU100, GLU143, ASN144, LEU146, ALA156, ASP157.
AICAR* (control)	-7.54	-6.20	
PPARα			
Soyasapogenol C	-8.95	-9.73	CYS276, THR279, LEU321, MET330, VAL332
Fenofibrate (control)	-9.67	-11.41	

- AICAR—5-Aminoimidazole-4-carboxamide ribonucleotide

Table S5. Human primer sets for real-time PCR.

Gene	Primer	
	Forward (5′→3′)	Reverse (3′→5′)
ACOX-1	AGGTCACAGCTGTCCAACCA	TTACCCAGCCCTGGCTTAAT
CPT-1α	GGTCCAGGTAGAGCTCAGGC	GTGCTCTGAGGCCTTTGTCA
ACC	GCTGCTCGGATCACTAGTGAA	TTCTGCTATCAGTCTGTCCAG
FASN	GACATCGTCCATTCGTTTGTG	CGGATCACCTTCTTGAGCTCC
GAPDH	CATGTTCGTCATGGGGTGAACCA	AGTGATGGCATGGACTGTGGTCAT
